# Advances in Automatic Software Verification: SV-COMP 2020

**DOI:** 10.1007/978-3-030-45237-7_21

**Published:** 2020-03-13

**Authors:** Dirk Beyer

**Affiliations:** 8 Johannes Kepler University, Linz, Austria; 9 University of Birmingham, Birmingham, UK; LMU Munich, Munich, Germany

**Keywords:** Formal Verification, Program Analysis, Competition

## Abstract

This report describes the 2020 Competition on Software Verification (SV-COMP), the 9$^{\text {th}}$ edition of a series of comparative evaluations of fully automatic software verifiers for C and Java programs. The competition provides a snapshot of the current state of the art in the area, and has a strong focus on replicability of its results. The competition was based on 11 052 verification tasks for C programs and 416 verification tasks for Java programs. Each verification task consisted of a program and a property (reachability, memory safety, overflows, termination). SV-COMP 2020 had 28 participating verification systems from 11 countries.

## 1 Introduction

The Competition on Software Verification (SV-COMP) serves as the showcase of the state of the art in the area of automatic software verification. SV-COMP 2020 is the 9th edition of the competition and presents an overview of the currently achieved results by tool implementations that are based on the most recent ideas, concepts, and algorithms for fully automatic verification. This competition report describes the (updated) rules and definitions, presents the competition results, and discusses some interesting facts about the execution of the competition experiments. The competition measures its own success by evaluating whether the objectives of the competition were achieved. To the objectives discussed earlier (1-4 [14]) we add two further objectives that deserve mentioning (5-6):

1. provide an overview of the state of the art in software-verification technology and increase visibility of the most recent software verifiers,
2. establish a repository of software-verification tasks that is publicly available for free use as standard benchmark suite for evaluating verification software,
3. establish standards that make it possible to compare different verification tools, including a property language and formats for the results,
4. accelerate the transfer of new verification technology to industrial practice by identifying the strengths of the various verifiers on a diverse set of tasks,
5. educate PhD students and others on performing replicable benchmarking, packaging tools, and running robust and accurate research experiments, and
6. provide research teams that do not have sufficient computing resources with the opportunity to obtain experimental results on large benchmark sets.

We now discuss the outcome of SV-COMP 2020 with respect to these objectives: (1) There were 28 participating software systems from 11 countries, using many different technologies (cf. Table 6). SV-COMP is considered an important event in the verification community. (2) The sv-benchmarks repository is considered one of the largest and most diverse collections of verification tasks in C and Java. The community dedicates a lot of maintenance effort, as the issue tracker [1] and the pull requests [2] on GitHub show. (3) SV-COMP has established a format for defining verification tasks, a standard specification language, and a set of functions to express non-deterministic values. Verification results are validated using verification witnesses and six different validators. (4) We received positive feedback from industry, reporting that it is helpful to look up the newest and best available verification tools, regarding the categories of interest. There are several participating systems from industry since 2017. (5) Participating in SV-COMP is also a challenge because the entry requirements are strict: the tools have to be packaged such that all necessary non-standard components are contained, the tools need to provide meaningful log output, the tool parameters have to be specified in the BenchExec benchmark-definition format, and a tool-info module needs to be implemented. All experiments are required to be fully replicable. It is a motivating experience to observe the learning of first-time participants. (6) Running large-scale performance experiments requires an infrastructure with considerable computing resources — which are not necessarily available to all tool developers. Through this competition and the preruns, the participants get the opportunity to repeatedly run experiments on the full benchmark set of verification tasks of the competition. The preruns and final run sum up to over one million verification runs and ten million witness-validation runs.

**Related Competitions.** It is well-understood that competitions are an important evaluation method, and there are many other competitions in the field of formal methods. The TOOLympics [3] [7] event in 2019 (part of the 25-years-of-TACAS celebration) presented 16 competitions in the area. Most closely related are the competitions RERS [4] [45] and VerifyThis [5] [46]. While SV-COMP [6] performs replicable experiments in a *controlled* environment (dedicated resources, resource limits), the RERS Challenges give more room for exploring combinations of interactive with automatic approaches without limits on the resources, and the VerifyThis Competition focuses on evaluating approaches and ideas rather than on *fully automatic* verification.

Large benchmark collections are extremely important to make approaches comparable and to agree on what constitutes interesting problems to solve. There are other large benchmark collections as well (e.g., by SPEC [7]), but the

[1] https://github.com/sosy-lab/sv-benchmarks/issues
[2] https://github.com/sosy-lab/sv-benchmarks/pulls
[3] https://tacas.info/toolympics.php
[4] http://rers-challenge.org
[5] http://etaps2016.verifythis.org
[6] https://sv-comp.sosy-lab.org
[7] https://www.spec.org

`sv-benchmarks` suite[8] is (a) free of charge, and (b) tailored to the state of the art in software verification. Benchmark repositories of various competitions and challenges also contribute to each other. For example, the `sv-benchmarks` suite contains programs that were originally used in RERS[9], in termCOMP[10], and in VerifyThis[11]. There is a flow of benchmarks in the other direction as well: The competition SMT-COMP [32] uses SMT formulas that were generated from programs of the `sv-benchmarks` collection. For example, the $k$-induction engine of CPAchecker was used to generate more than 1000 SMT formulas for the quantifier-free theory of arrays and bit-vectors (QF_ABV)[12].

## 2 Organization, Definitions, Formats, and Rules

**Procedure.** SV-COMP 2020's overall organization did not change in comparison to the earlier editions [8, 9, 10, 11, 12, 13, 14]. SV-COMP is an open competition, where all verification tasks are known before the submission of the participating verifiers, which is necessary due to the complexity of the C language. During the *benchmark submission* phase, new verification tasks were collected, classified, and added to the existing benchmark suite (i.e., SV-COMP uses an accumulating benchmark suite), during the *training* phase, the teams inspected the verification tasks and trained their verifiers (also, the verification tasks received fixes and quality improvement), and during the *evaluation* phase, verification runs were preformed with all competition candidates, and the system descriptions and archives were reviewed by the competition jury. The participants received the results of their verifier directly via e-mail, and after a few days of inspection, the results were publicly announced on the competition web site. The *Competition Jury* consisted again of the chair and one member of each participating team. Team representatives of the jury are listed in Table 5.

**Qualification and License Requirements.** As a new feature in SV-COMP 2020, a rule was introduced that allows the organizer to reuse systems that participated in previous years, and to enter new systems, provided that the developers were given the chance to contribute a submission themselves (both options were not used this time). Starting 2018, SV-COMP required that the verifier must be publicly available for download and has a license that

(i) allows replication and evaluation by anybody (including results publication),
(ii) does not restrict the usage of the verifier output (log files, witnesses), and
(iii) allows any kind of (re-)distribution of the unmodified verifier archive.

8 https://github.com/sosy-lab/sv-benchmarks
9 https://github.com/sosy-lab/sv-benchmarks/blob/svcomp20/c/eca-rers2012/README.txt
10 https://github.com/sosy-lab/sv-benchmarks/blob/svcomp20/c/termination-restricted-15/README.txt
11 https://github.com/sosy-lab/sv-benchmarks/blob/svcomp20/c/verifythis/README.txt
12 https://clc-gitlab.cs.uiowa.edu:2443/SMT-LIB-benchmarks-inc/QF_ABV/tree/master/20190307-CPAchecker_kInduction-SoSy_Lab

```
1  format_version: '1.0'
2
3  # old file name: floppy_true-unreach-call_true-valid-memsafety.i.cil.c
4  input_files: 'floppy.i.cil-3.c'
5
6  properties:
7    - property_file: ../properties/unreach-call.prp
8      expected_verdict: true
9    - property_file: ../properties/valid-memsafety.prp
10     expected_verdict: false
11     subproperty: valid-memtrack
```

Fig. 1: Example task definition for program `floppy.i.cil-3.c`

**Validation of Results.** The validation of the results based on verification witnesses [19, 20] was done as in previous years (2017–2019), mandatory for *both* answers True or False. A few categories were excluded from validation if the validators did not sufficiently support a certain kind of program or property. Two new validators participated in SV-COMP 2020: Nitwit [66] and MetaVal [25].

**Verification Tasks — Explicit Task-Definition Files.** The notion of verification tasks did not change and we refer to previous reports for more details [10, 13]. We developed a new format for task definitions that was used for the Java category already in SV-COMP 2019. Technically, we need a verification task (a pair of a program and a specification to verify) to feed as input to the verifier, and an expected result against which we check the answer that the verifier returns. Previously, the above-mentioned three components were specified in the file name of the program; now all the information is stored in an extra file that contains a structured definition of the verification tasks for a program. For each program, the repository contains the program file and a task-definition file. Consider an example program that is available under the name `floppy.i.cil-3.c`: This program comes now with its task-definition file `floppy.i.cil-3.yml`. Figure 1 shows this task definition. The new format was used in SV-COMP 2019 for the Java category [14] and in the competition on software testing, Test-Comp 2019 [15].

The task definition uses the YAML format as underlying structured data format. It contains a version id of the format (line 1) and can contain comments (line 3). The field `input_files` specifies the input program (example: '`floppy.i.cil-3.c`'), which is either one file or a list of files. The field `properties` lists all properties of the specification for this program. Each property has a field `property_file` that specifies the property file (example: `../properties/unreach-call.prp`) and a field `expected_verdict` that specifies the expected result (example: `true`).

**Categories, Properties, Scoring Schema, and Ranking.** The categories are listed in Tables 7 and 8 and described in detail on the competition web site.[13] Figure 2 shows the category composition. For the definition of the properties and the property format, we refer to the 2015 competition report [11]. All specifications are available in the directory `c/properties/` of the benchmark

[13] https://sv-comp.sosy-lab.org/2020/benchmarks.php

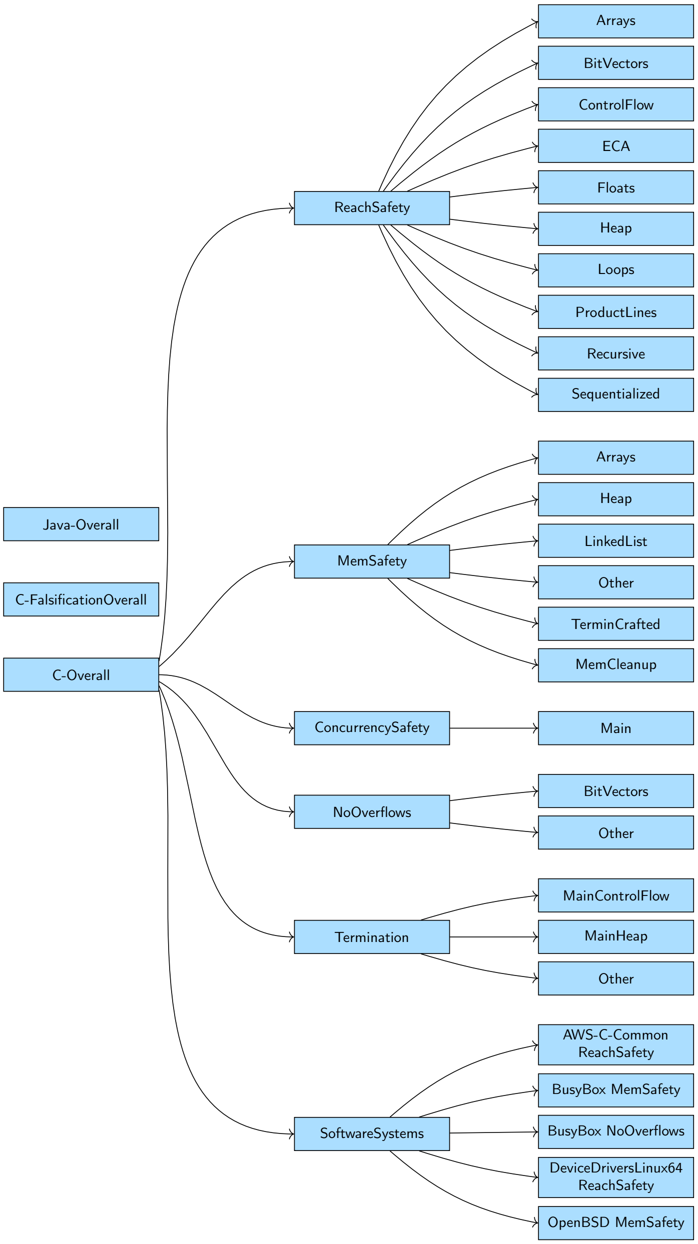

Fig. 2: Category structure for SV-COMP 2020; category *C-FalsificationOverall* contains all verification tasks of *C-Overall* without *Termination*; *Java-Overall* contains all Java verification tasks

Table 1: Properties used in SV-COMP 2020 (unchanged since 2019 [14])

| Formula | Interpretation |
|---|---|
| `G ! call(foo())` | A call to function `foo` is not reachable on any finite execution. |
| `G valid-free` | All memory deallocations are valid (counterexample: invalid free). More precisely: There exists no finite execution of the program during which an invalid memory deallocation occurs. |
| `G valid-deref` | All pointer dereferences are valid (counterexample: invalid dereference). More precisely: There exists no finite execution of the program during which an invalid pointer dereference occurs. |
| `G valid-memtrack` | All allocated memory is tracked, i.e., pointed to or deallocated (counterexample: memory leak). More precisely: There exists no finite execution of the program during which the program lost track of some previously allocated memory. |
| `G valid-memcleanup` | All allocated memory is deallocated before the program terminates. In addition to valid-memtrack: There exists no finite execution of the program during which the program terminates but still points to allocated memory. (Comparison to Valgrind: This property can be violated even if Valgrind reports 'still reachable'.) |
| `F end` | All program executions are finite and end on proposition `end`, which marks all program exits (counterexample: infinite loop). More precisely: There exists no execution of the program on which the program never terminates. |

Table 2: Scoring schema for SV-COMP 2020 (unchanged since 2017 [13])

| Reported result | Points | Description |
|---|---|---|
| Unknown | 0 | Failure to compute verification result |
| False correct | +1 | Violation of property in program was correctly found and a validator confirmed the result based on a witness |
| False incorrect | −16 | Violation reported but property holds (false alarm) |
| True correct | +2 | Program correctly reported to satisfy property and a validator confirmed the result based on a witness |
| True correct unconfirmed | +1 | Program correctly reported to satisfy property, but the witness was not confirmed by a validator |
| True incorrect | −32 | Incorrect program reported as correct (wrong proof) |

repository. Table 1 lists the properties and their syntactical representation as overview. Property `G valid-memcleanup`, and thus, the category *MemCleanup*, was used for the first time in SV-COMP 2019. The categories *AWS-C-Common* and *OpenBSD* were added for SV-COMP 2020.

The scoring schema is identical for SV-COMP 2017–2020: Table 2 provides the overview and Fig. 3 visually illustrates the score assignment for one property. The scoring schema still contains the special rule for unconfirmed correct results for expected result True that was introduced in the transitioning phase: one point is assigned if the answer matches the expected result but the witness was not confirmed.

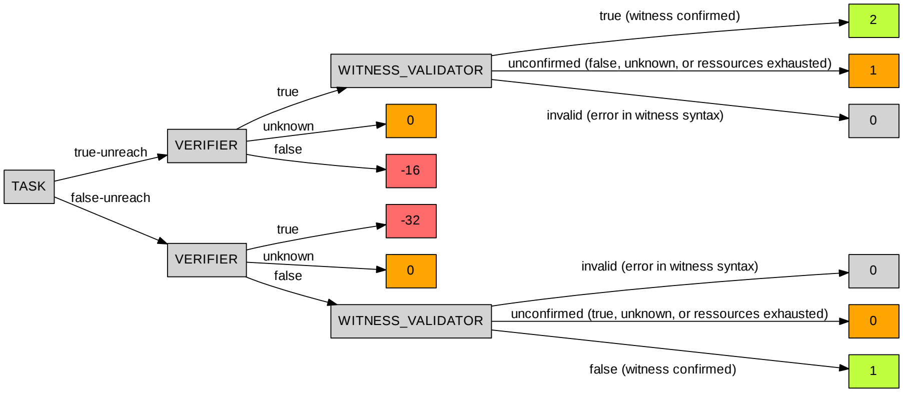

Fig. 3: Visualization of the scoring schema for the reachability property (from [13], © Springer-Verlag)

The ranking was again decided based on the sum of points (normalized for meta categories). In case of a tie, the ranking was decided based on success run time, which is the total CPU time over all verification tasks for which the verifier reported a correct verification result. *Opt-out from Categories* and *Score Normalization for Meta Categories* was done as described previously [9] (page 597).

## 3 Reproducibility

All major components used in the competition are available in public version repositories. This allows independent replication of the SV-COMP experiments. An overview of the components that contribute to the reproducible setup of SV-COMP is provided in Fig. 4, and the details are given in Table 3. The SV-COMP 2016 report [12] describes all components of the SV-COMP organization and how we ensure that all parts are publicly available for maximal replicability.

We have published the competition artifacts at Zenodo to guarantee their long-term availability and immutability. These artifacts comprise the verification tasks, the produced competition results, and the produced verification witnesses. The DOIs and references are given in Table 4. The archive for the competition results includes the raw results in BenchExec's XML exchange format, the log output of the verifiers and validators, and a mapping from files names to SHA-256 hashes. The hashes of the files are useful for validating the exact contents of a file, and accessing the files inside the archive that contains the verification witnesses.

To provide a more transparent way of accessing the exact versions of the verifiers that were used in the competition, all verifier archives are stored in a public Git repository. GitLab was used to host the repository for the verifier archives due to its generous repository size limit of 10 GB. The final size of the Git repository is 5.78 GB.

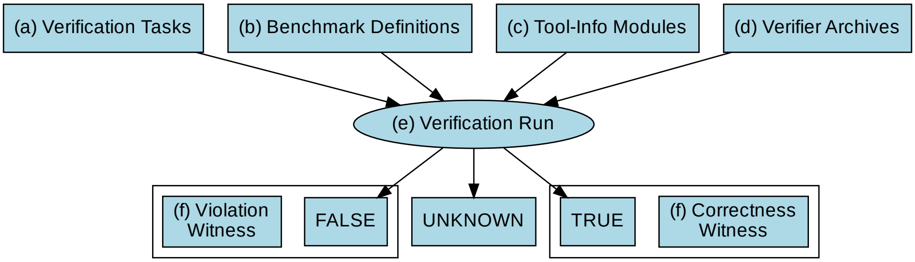

Fig. 4: SV-COMP components and the execution flow

Table 3: Publicly available components for replicating SV-COMP 2020

| Component | Fig. 4 | Repository | Version |
|---|---|---|---|
| Verification Tasks | (a) | github.com/sosy-lab/sv-benchmarks | svcomp20 |
| Benchmark Definitions | (b) | github.com/sosy-lab/sv-comp | svcomp20 |
| Tool-Info Modules | (c) | github.com/sosy-lab/benchexec | 2.5.1 |
| Verifier Archives | (d) | gitlab.com/sosy-lab/sv-comp/archives-2020 | svcomp20 |
| Benchmarking | (e) | github.com/sosy-lab/benchexec | 2.5.1 |
| Witness Format | (f) | github.com/sosy-lab/sv-witnesses | svcomp20 |

## 4 Results and Discussion

The results of the competition experiments represent the state of the art in fully automatic software-verification tools. The report shows the results, in terms of effectiveness (number of verification tasks that can be solved and correctness of the results, as accumulated in the score) and efficiency (resource consumption in terms of CPU time). The results are presented in the same way as in last years, such that the improvements compared to last year are easy to identify. The results presented in this report were inspected and approved by the participating teams. We now discuss the highlights of the results.

**Participating Verifiers.** Table 5 and the competition web site[14] provide an overview of the participating verification systems. Table 6 lists the algorithms and techniques that are used in the verification tools.

**Computing Resources.** The resource limits were the same as in the previous competitions [12]: Each verification run was limited to 8 processing units (cores), 15 GB of memory, and 15 min of CPU time. The witness validation was limited to 2 processing units, 7 GB of memory, and 1.5 min of CPU time for violation witnesses and 15 min of CPU time for correctness witnesses. The machines for running the experiments are part of a compute cluster that consists of

[14] https://sv-comp.sosy-lab.org/2020/systems.php

Table 4: Artifacts published for SV-COMP 2020

| Content | DOI | Reference |
|---|---|---|
| Verification Tasks | 10.5281/zenodo.3633334 | [17] |
| Competition Results | 10.5281/zenodo.3630205 | [16] |
| Verification Witnesses | 10.5281/zenodo.3630188 | [18] |

Table 5: Competition candidates with tool references and representing jury members

| **Participant** | **Ref.** | **Jury member** | **Affiliation** |
|---|---|---|---|
| 2LS | [26, 55] | Viktor Malík | BUT, Brno, Czechia |
| Brick | | Lei Bu | Nanjing U., China |
| Cbmc | [51] | Michael Tautschnig | Amazon Web Services, UK |
| Coastal | [67] | Willem Visser | Stellenbosch U., South Africa |
| CPA-BAM-BnB | [3, 68] | Vadim Mutilin | ISP RAS, Russia |
| CPA-Lockator | [4] | Pavel Andrianov | ISP RAS, Russia |
| CPA-Seq | [22, 36] | Martin Spiessl | LMU Munich, Germany |
| Dartagnan | [40, 53] | Hernán Ponce de León | Bundeswehr U. Munich, Germany |
| Divine | [6, 52] | Henrich Lauko | Masaryk U., Czechia |
| Esbmc | [38, 39] | Felipe R. Monteiro | Federal U. of Amazonas, Brazil |
| Gacal | [61] | Benjamin Quiring | Northeastern U., USA |
| Java-Ranger | [65] | Vaibhav Sharma | U. of Minnesota, USA |
| JayHorn | [49, 50] | Philipp Ruemmer | Uppsala U., Sweden |
| JBmc | [33, 34] | Peter Schrammel | U. of Sussex, UK |
| JDart | [54, 56] | Falk Howar | TU Dortmund, Germany |
| Lazy-CSeq | [47, 48] | Omar Inverso | Gran Sasso Science Inst., Italy |
| Map2Check | [63, 64] | Herbert Rocha | Federal U. of Roraima, Brazil |
| PeSCo | [35, 62] | Cedric Richter | Paderborn U., Germany |
| Pinaka | [30] | Saurabh Joshi | IIT Hyderabad, India |
| PredatorHP | [44, 59] | Veronika Šoková | BUT, Brno, Czechia |
| SPF | [57, 60] | Willem Visser | Amazon, USA |
| Symbiotic | [28, 29] | Marek Chalupa | Masaryk U., Czechia |
| UAutomizer | [42, 43] | Matthias Heizmann | U. of Freiburg, Germany |
| UKojak | [27, 58] | Alexander Nutz | U. of Freiburg, Germany |
| UTaipan | [37, 41] | Daniel Dietsch | U. of Freiburg, Germany |
| VeriAbs | [1, 2] | Priyanka Darke | Tata Consultancy Services, India |
| VeriFuzz | [31] | Raveendra Kumar M. | Tata Consultancy Services, India |
| Yogar-Cbmc | [70, 71] | Liangze Yin | Nat. U. of Defense Techn., China |

168 machines; each verification run was executed on an otherwise completely unloaded, dedicated machine, in order to achieve precise measurements. Each machine had one Intel Xeon E3-1230 v5 CPU, with 8 processing units each, a frequency of 3.4 GHz, 33 GB of RAM, and a GNU/Linux operating system (x86_64-linux, Ubuntu 18.04 with Linux kernel 4.15). We used BenchExec [23] to measure and control computing resources (CPU time, memory, CPU energy) and VerifierCloud [15] to distribute, install, run, and clean-up verification runs,

[15] https://vcloud.sosy-lab.org

Table 6: Algorithms and techniques that the competition candidates offer

| Participant | CEGAR | Predicate Abstraction | Symbolic Execution | Bounded Model Checking | k-Induction | Property-Directed Reach. | Explicit-Value Analysis | Numeric. Interval Analysis | Shape Analysis | Separation Logic | Bit-Precise Analysis | ARG-Based Analysis | Lazy Abstraction | Interpolation | Automata-Based Analysis | Concurrency Support | Ranking Functions | Evolutionary Algorithms |
|---|---|---|---|---|---|---|---|---|---|---|---|---|---|---|---|---|---|---|
| 2LS | | | | ✓ | ✓ | | | ✓ | ✓ | | ✓ | | | | | | ✓ | |
| Brick | ✓ | | ✓ | ✓ | | | | ✓ | | | | | | | | ✓ | | |
| Cbmc | | | | ✓ | | | | | | | ✓ | | | | | ✓ | | |
| Coastal | | | ✓ | | | | | | | | | | | | | | | |
| CPA-BAM-BnB | ✓ | ✓ | | | | | ✓ | | | | ✓ | ✓ | ✓ | ✓ | | | | |
| CPA-Lockator | ✓ | ✓ | | | | | ✓ | | | | ✓ | ✓ | ✓ | ✓ | | ✓ | | |
| CPA-Seq | ✓ | ✓ | | ✓ | ✓ | | ✓ | ✓ | ✓ | | ✓ | ✓ | ✓ | ✓ | | ✓ | ✓ | |
| Dartagnan | | | | ✓ | | | | | | | | | | | | ✓ | | |
| Divine | | | ✓ | | | | ✓ | | | | ✓ | | | | | ✓ | | |
| Esbmc | | | | ✓ | ✓ | | | | | | ✓ | | | | | ✓ | | |
| Gacal | | | | | | | | | | | | | | | | | | |
| Java-Ranger | | | ✓ | | | | | | | | ✓ | | | | | | | |
| JayHorn | ✓ | ✓ | | | | ✓ | | ✓ | | | | | ✓ | ✓ | | | | |
| JBmc | | | | ✓ | | | | | | | ✓ | | | | | ✓ | | |
| JDart | | | ✓ | | | | | | | | ✓ | | | | | | | |
| Lazy-CSeq | | | | ✓ | | | | | | | ✓ | | | | | ✓ | | |
| Map2Check | | | | ✓ | | | | | | | ✓ | | | | | | | |
| PeSCo | ✓ | ✓ | | ✓ | ✓ | | ✓ | ✓ | ✓ | | ✓ | ✓ | ✓ | ✓ | | ✓ | ✓ | |
| Pinaka | | | ✓ | ✓ | | | | | | | ✓ | | | | | | | |
| PredatorHP | | | | | | | | | ✓ | | | | | | | | | |
| SPF | | | ✓ | | | | | | ✓ | | | | | | | ✓ | | |
| Symbiotic | | | ✓ | | | | | ✓ | ✓ | | ✓ | | | | | | | |
| UAutomizer | ✓ | ✓ | | | | | | | | | ✓ | | ✓ | ✓ | ✓ | ✓ | ✓ | |
| UKojak | ✓ | ✓ | | | | | | | | | ✓ | | ✓ | ✓ | | | | |
| UTaipan | ✓ | ✓ | | | | | ✓ | ✓ | | | ✓ | | ✓ | ✓ | ✓ | ✓ | | |
| VeriAbs | ✓ | | | ✓ | ✓ | | ✓ | ✓ | | | | | | | | | | ✓ |
| VeriFuzz | | | | ✓ | | | | ✓ | | | | | | | | | | ✓ |
| Yogar-Cbmc | ✓ | | | ✓ | | | | | | | ✓ | | ✓ | | | ✓ | | |

and to collect the results. The values for time and energy are accumulated over all cores of the CPU. To measure the CPU energy, we use CPU Energy Meter [24] (integrated in BenchExec [23]).

One complete verification execution of the competition consisted of 138 074 verification runs (each verifier on each verification task of the selected categories according to the opt-outs), consuming 491 days of CPU time and 130 kWh of CPU energy (without validation). Witness-based result validation required 684 858 validation runs (each validator on each verification task for categories with witness validation, and for each verifier), consuming 311 days of CPU time. Each tool was executed several times, in order to make sure no installation issues occur during the execution. Including preruns, the infrastructure managed a total of 1 018 781 verification runs consuming 4.8 years of CPU time, and 10 705 227 validation runs consuming 6.9 years of CPU time.

**Quantitative Results.** Table 7 presents the quantitative overview of all tools and all categories. The head row mentions the category, the maximal score for the category, and the number of verification tasks. The tools are listed in alphabetical order; every table row lists the scores of one verifier. We indicate the top three candidates by formatting their scores in bold face and in larger font size. An empty table cell means that the verifier opted-out from the respective main category (perhaps participating in subcategories only, restricting the evaluation to a specific topic). More information (including interactive tables, quantile plots for every category, and also the raw data in XML format) is available on the competition web site [16] and in the results artifact (see Table 4).

Table 8 reports the top three verifiers for each category. The run time (column 'CPU Time') and energy (column 'CPU Energy') refer to successfully solved verification tasks (column 'Solved Tasks'). We also report the number of tasks for which no witness validator was able to confirm the result (column 'Unconf. Tasks'). The columns 'False Alarms' and 'Wrong Proofs' report the number of verification tasks for which the verifier reported wrong results, i.e., reporting a counterexample when the property holds (incorrect False) and claiming that the program fulfills the property although it actually contains a bug (incorrect True), respectively.

**Score-Based Quantile Functions for Quality Assessment.** We use score-based quantile functions [9, 23] because these visualizations make it easier to understand the results of the comparative evaluation. The web site [16] and the results archive (see Table 4) include such a plot for each category. As an example, we show the plot for category *C-Overall* (all verification tasks) in Fig. 5. A total of 11 verifiers participated in category *C-Overall*, for which the quantile plot shows the overall performance over all categories (scores for meta categories are normalized [9]). A more detailed discussion of score-based quantile plots, including examples of what insights one can obtain from the plots, is provided in previous competition reports [9, 12].

[16] https://sv-comp.sosy-lab.org/2020/results

Table 7: Quantitative overview over all results; empty cells represent opt-outs

| **Participant** | **ReachSafety** 6681 points 4079 tasks | **MemSafety** 809 points 512 tasks | **ConcurrencySafety** 1344 points 1082 tasks | **NoOverflows** 612 points 397 tasks | **Termination** 3563 points 2043 tasks | **SoftwareSystems** 4879 points 2939 tasks | **FalsificationOverall** 4211 points 9009 tasks | **Overall** 17328 points 11052 tasks | **JavaOverall** 602 points 416 tasks |
|---|---|---|---|---|---|---|---|---|---|
| 2LS | 2491 | 298 | 0 | 340 | **1264** | 13 | 914 | 4924 | |
| Brick | | | | | | | | | |
| Cbmc | 2864 | -162 | 554 | 268 | 499 | 30 | 1256 | 3365 | |
| CPA-BAM-BnB | | | | | | **602** | | | |
| CPA-Seq | **4396** | **355** | **996** | **483** | **1720** | **746** | **2772** | **9219** | |
| CPA-Lockator | | | -387 | | | | | | |
| Dartagnan | | | 173 | | | | | | |
| Divine | -76 | 71 | 550 | 0 | 0 | -12 | 585 | 1151 | |
| Esbmc | 3481 | 334 | 325 | 264 | 777 | 500 | **1639** | 5567 | |
| Gacal | | | | | | | | | |
| Lazy-CSeq | | | **1279** | | | | | | |
| Map2Check | | -68 | | -89 | | | | | |
| PeSCo | **4376** | | | | | | 1590 | **8023** | |
| Pinaka | 2585 | | | 243 | 590 | | | | |
| PredatorHP | | **611** | | | | | | | |
| Symbiotic | 2753 | **516** | 0 | 294 | 1022 | **954** | **1828** | 5985 | |
| UAutomizer | 2696 | 354 | 296 | **466** | **2942** | 591 | 893 | **8178** | |
| UKojak | 1702 | 231 | 0 | 387 | 0 | 501 | 1148 | 3710 | |
| UTaipan | 2185 | 316 | 289 | **461** | 0 | 482 | 805 | 5057 | |
| VeriAbs | **5543** | 0 | 0 | 0 | 0 | 244 | 273 | 2656 | |
| VeriFuzz | 1206 | | | 146 | | | | | |
| Yogar-Cbmc | | | **1275** | | | | | | |
| Coastal | | | | | | | | | 472 |
| Java-Ranger | | | | | | | | | **549** |
| JayHorn | | | | | | | | | 278 |
| JBmc | | | | | | | | | **527** |
| JDart | | | | | | | | | **524** |
| SPF | | | | | | | | | 410 |

Table 8: Overview of the top-three verifiers for each category (measurement values for CPU time and energy rounded to two significant digits)

| Rank | Verifier | Score | CPU Time (in h) | CPU Energy (in kWh) | Solved Tasks | Unconf. Tasks | False Alarms | Wrong Proofs |
|---|---|---|---|---|---|---|---|---|
| ***ReachSafety*** | | | | | | | | |
| 1 | **VeriAbs** | **5543** | 150 | 1.6 | 3 412 | 171 | | |
| 2 | CPA-Seq | 4396 | 72 | .75 | 2 700 | 54 | 8 | |
| 3 | PeSCo | 4376 | 39 | .38 | 2 518 | 36 | 4 | |
| ***MemSafety*** | | | | | | | | |
| 1 | **PredatorHP** | **611** | .78 | .010 | 392 | 15 | | |
| 2 | Symbiotic | 516 | .51 | .010 | 358 | 6 | | |
| 3 | CPA-Seq | 355 | .76 | .010 | 264 | 1 | | |
| ***ConcurrencySafety*** | | | | | | | | |
| 1 | **Lazy-CSeq** | **1279** | 6.7 | .090 | 1 023 | 44 | | |
| 2 | Yogar-Cbmc | 1275 | .39 | .000 | 1 024 | 33 | | |
| 3 | CPA-Seq | 996 | 12 | .11 | 830 | 102 | | |
| ***NoOverflows*** | | | | | | | | |
| 1 | **CPA-Seq** | **483** | .93 | .010 | 321 | 8 | | |
| 2 | UAutomizer | 466 | 1.4 | .010 | 326 | 0 | | |
| 3 | UTaipan | 461 | 1.5 | .010 | 323 | 0 | | |
| ***Termination*** | | | | | | | | |
| 1 | **UAutomizer** | **2942** | 15 | .16 | 1 606 | 7 | | |
| 2 | CPA-Seq | 1720 | 16 | .17 | 1 247 | 7 | | |
| 3 | 2ls | 1264 | 3.2 | .030 | 955 | 361 | 3 | |
| ***SoftwareSystems*** | | | | | | | | |
| 1 | **Symbiotic** | **954** | .25 | .000 | 676 | 36 | 3 | **1** |
| 2 | CPA-Seq | 746 | 21 | .24 | 1 381 | 363 | 1 | |
| 3 | CPA-BAM-BnB | 602 | 8.0 | .070 | 1 411 | 582 | 3 | **4** |
| ***FalsificationOverall*** | | | | | | | | |
| 1 | **CPA-Seq** | **2772** | 45 | .45 | 2 240 | 139 | 9 | |
| 2 | Symbiotic | 1828 | 27 | .35 | 1 461 | 10 | 3 | |
| 3 | Esbmc | 1639 | 14 | .18 | 1 819 | 385 | 16 | |
| ***Overall*** | | | | | | | | |
| 1 | **CPA-Seq** | **9219** | 120 | 1.3 | 6 743 | 535 | 9 | |
| 2 | UAutomizer | 8178 | 83 | .84 | 5 523 | 693 | 71 | **2** |
| 3 | PeSCo | 8023 | 120 | 1.2 | 6 402 | 242 | 32 | |
| ***JavaOverall*** | | | | | | | | |
| 1 | **Java-Ranger** | **549** | 1.3 | .010 | 376 | | | |
| 2 | JBmc | 527 | .18 | .000 | 376 | | | |
| 3 | JDart | 524 | .26 | .000 | 374 | | | |

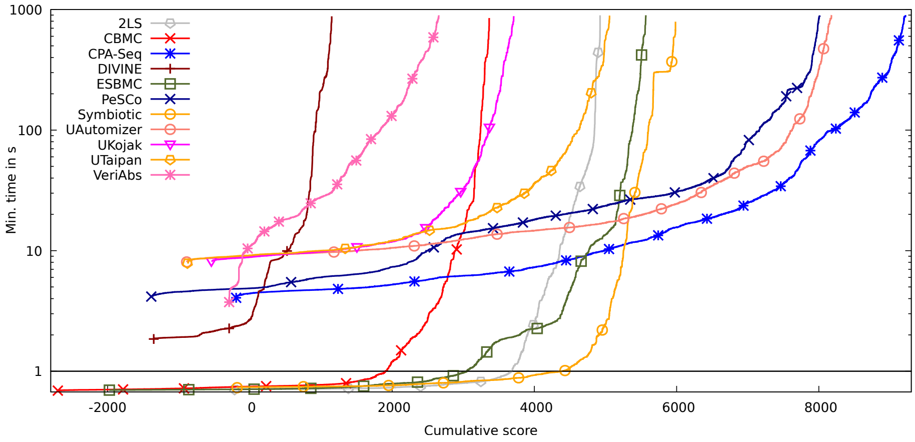

Fig. 5: Quantile functions for category *C-Overall*. Each quantile function illustrates the quantile ($x$-coordinate) of the scores obtained by correct verification runs below a certain run time ($y$-coordinate). More details were given previously [9]. A logarithmic scale is used for the time range from 1 s to 1000 s, and a linear scale is used for the time range between 0 s and 1 s.

**Alternative Rankings.** The community suggested to report a couple of alternative rankings that honor different aspects of the verification process as complement to the official SV-COMP ranking. Table 9 is similar to Table 8, but contains the alternative ranking categories *Correct* and *Green Verifiers*. Column 'Quality' gives the score in score points, column 'CPU Time' the CPU usage of successful runs in hours, column 'CPU Energy' the CPU usage of successful runs in kWh, column 'Solved Tasks' the number of correct results, column 'Wrong Results' the sum of false alarms and wrong proofs in number of errors, and column 'Rank Measure' gives the measure to determine the alternative rank.

*Correct Verifiers — Low Failure Rate.* The right-most columns of Table 8 report that the verifiers achieve a high degree of correctness (all top three verifiers in the C track have less than 2 % wrong results). The winners of category *Java-Overall* produced not a single wrong answer. The first category in Table 9 uses a failure rate as rank measure: $\frac{\text{number of incorrect results}}{\text{total score}}$, the number of errors per score point ($E/sp$). We use $E$ as unit for number of incorrect results and $sp$ as unit for total score. It is remarkable to see that the worst result was 0.38 E/sp in SV-COMP 2019 and is now improved to 0.032 E/sp, with is an order of magnitude better.

*Green Verifiers — Low Energy Consumption.* Since a large part of the cost of verification is given by the energy consumption, it might be important to also consider the energy efficiency. The second category in Table 9 uses the energy consumption per score point as rank measure: $\frac{\text{total CPU energy}}{\text{total score}}$, with the unit $J/sp$. It is interesting to see that the worst result from SV-COMP 2019 was 4 200 J/sp, and now it is improved to 2 200 J/sp.

Table 9: Alternative rankings; quality is given in score points (sp), CPU time in hours (h), energy in kilojoule (kJ), wrong results in errors (E), rank measures in errors per score point (E/sp), joule per score point (J/sp), and score points (sp)

| Rank | Verifier | Quality (sp) | CPU Time (h) | CPU Energy (kWh) | Solved Tasks | Wrong Results (E) | Rank Measure |
|---|---|---|---|---|---|---|---|
| ***Correct Verifiers*** | | | | | | | (E/sp) |
| 1 | **CPA-Seq** | 9 219 | 120 | 1.3 | 6 743 | 9 | .0010 |
| 2 | UKojak | 3 710 | 48 | 0.49 | 2 405 | 4 | .0011 |
| 3 | 2ls | 4 924 | 27 | 0.24 | 3 044 | 8 | .0016 |
| worst | | | | | | | .032 |
| ***Green Verifiers*** | | | | | | | (J/sp) |
| 1 | **Cbmc** | 3 365 | 15 | 0.16 | 3 217 | 67 | 170 |
| 2 | 2ls | 4 924 | 27 | 0.24 | 3 044 | 8 | 180 |
| 3 | Esbmc | 5 567 | 35 | 0.41 | 5 520 | 51 | 270 |
| worst | | | | | | | 2 200 |

Table 10: Confirmation rate of verification witnesses in SV-COMP 2020

| Result | True | | | | False | | | |
|---|---|---|---|---|---|---|---|---|
| | Total | Confirmed | | Unconf. | Total | Confirmed | | Unconf. |
| 2ls | 2 060 | 2 049 | 99 % | 11 | 1 449 | 995 | 69 % | 454 |
| Cbmc | 1 949 | 1 821 | 93 % | 128 | 2 095 | 1 396 | 67 % | 699 |
| CPA-Seq | 4 347 | 3 958 | 91 % | 389 | 2 931 | 2 785 | 95 % | 146 |
| Divine | 811 | 793 | 98 % | 18 | 1 099 | 672 | 61 % | 427 |
| Esbmc | 3 779 | 3 701 | 98 % | 78 | 2 204 | 1 819 | 83 % | 385 |
| PeSCo | 3 777 | 3 704 | 98 % | 73 | 2 867 | 2 698 | 94 % | 169 |
| Symbiotic | 2 196 | 2 146 | 98 % | 50 | 1 996 | 1 879 | 94 % | 117 |
| UAutomizer | 4 135 | 4 029 | 97 % | 106 | 2 081 | 1 494 | 72 % | 587 |
| **UKojak** | 1 811 | 1 801 | 99 % | **10** | 606 | 604 | 100 % | **2** |
| UTaipan | 2 496 | 2 438 | 98 % | 58 | 1 308 | 730 | 56 % | 578 |
| VeriAbs | 3 908 | 3 387 | 87 % | 521 | 1 536 | 1 332 | 87 % | 204 |

**Verifiable Witnesses.** All SV-COMP verifiers are required to justify the result (True or False) by producing a verification witness (except for those categories for which no witness validator is available). We used six independently developed witness-based result validators [19, 20, 21, 25, 66].

The majority of witnesses that the verifiers produced can be confirmed by the results-validation process. Interestingly, the confirmation rate for the True results is significantly higher than for the False results. Table 10 shows the confirmed versus unconfirmed results: the first column lists the verifiers

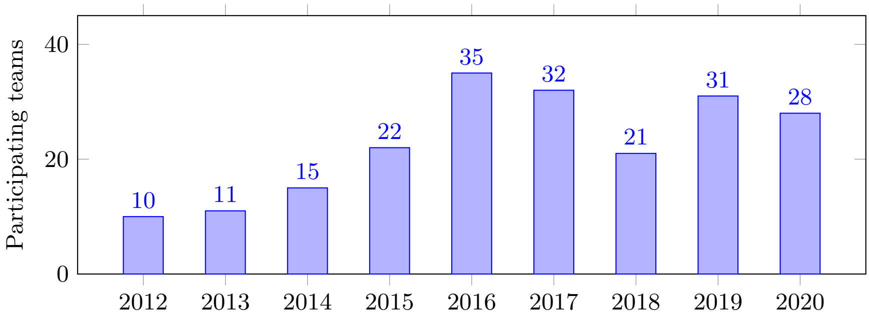

Fig. 6: Number of participating teams for each year of category *C-Overall*, the three columns for result TRUE reports the total, confirmed, and unconfirmed number of verification tasks for which the verifier answered with TRUE, respectively, and the three columns for result FALSE reports the total, confirmed, and unconfirmed number of verification tasks for which the verifier answered with FALSE, respectively. More information (for all verifiers) is given in the detailed tables on the competition web site [16] and in the results artifact; all verification witnesses are also contained in the witnesses artifact (see Table 4). Result validation is an important topic also in other competitions (e.g., in the SAT competition [5, 69]).

## 5 Conclusion

SV-COMP 2020, the 9th edition of the Competition on Software Verification, attracted 28 participating teams from 11 countries (see Fig. 6 for the participation numbers). SV-COMP continues to offer a broad overview of the state of the art in automatic software verification. The competition does not only execute the verifiers and collect results, but also validates the verification results, using six independently developed results validators. The number of verification tasks was increased to 11 052 in C and to 416 in Java. As before, the large jury and the organizer made sure that the competition follows the high quality standards of the TACAS conference, in particular with respect to the important principles of fairness, community support, and transparency.

**Data Availability Statement.** The verification tasks and results of the competition are published at Zenodo, as described in Table 4. All components and data that are necessary for reproducing the competition are available in public version repositories, as specified in Fig. 4 and Table 3. Furthermore, the results are presented online on the competition web site for easy access: https://sv-comp.sosy-lab.org/2020/results/.
